# Replacement of fish meal with full fat *Hermetia illucens* modulates hepatic FXR signaling in juvenile rainbow trout (*Oncorhynchus mykiss*): Exploring a potential role of ecdysteroids

**DOI:** 10.1016/j.heliyon.2024.e40302

**Published:** 2024-11-08

**Authors:** Paolo Cocci, Mauro Angeletti, Gilberto Mosconi, Ike Olivotto, Matteo Zarantoniello, Francesco Alessandro Palermo

**Affiliations:** a University of Camerino, School of Biosciences and Veterinary Medicine, Camerino, 62032, Italy; b Polytechnic University of Marche, Department of Life and Environmental Sciences, Ancona, 60131, Italy

**Keywords:** 20-Hydroxyecdysone, Farnesoid X receptor, Nuclear receptors, Bile acids

## Abstract

The present study was conducted to investigate the effects of fish meal (FM) replacement with full fat *Hermetia illucens* (HI) on the molecular mechanisms regulating lipid and bile salt (BA) homeostasis in rainbow trout (*Oncorhynchus mykiss*) juveniles. We thus explore the presence of 20-hydroxyecdysone (20E) in an insect meal-based diet and evaluate its potential involvement in regulating the molecular mechanisms/basis of FXR:RXR axis signaling. Ecdysteroids are a category of steroid hormones which bind a nuclear–receptor complex composed of ecdysone receptor (EcR) and ultraspiracle protein (USP) and regulate insect molting and metamorphosis. In all vertebrates, including fish, EcR-USP homologs are the Farnesoid X receptors (FXR) and the Retinoid X receptors (RXR), which are known to regulate crucial physiological and metabolic aspects, including BA synthesis and cholesterol homeostasis. *In silico* prediction indicates that 20E binds the heterodimeric complex with a binding affinity constant K_d_ equals to 610 ± 60 nM and affects positively the dimerization process. Results also demonstrated the coordinated increased expression of FXR and RXR, as well as their downstream target genes (*i.e.* short heterodimer partner 1 and 2) in rainbow trout fed diets containing HI meal. This latter finding was paralleled by a significant down-regulation of CYP7a1 and CYP8b1 gene expression together with a decrease in hepatic total cholesterol, triglyceride, and BA levels. Overall, our study suggested that FXR is a potential target for 20E content in insect meal and provided preliminary data on the potential role of ecdysteroids in regulating the metabolic status of teleost fish through modulation of FXR signaling in the enterohepatic system.

## 1 Introduction

It has been well established that insect molting and metamorphosis shows an endocrine control that is exerted by a category of steroid hormones known as ecdysteroids [1]. One of the main insect molting hormones is the ecdysone, primarily synthesized by the prothoracic gland, that is converted to 20-hydroxyecdysone (20E; a biologically active ecdysteroid) in peripheral tissues [2]. In the ecdysone pathway, 20E binds to a nuclear–receptor complex composed of ecdysone receptor (EcR) and ultraspiracle protein (USP). The EcR-USP heterodimer triggers a transcriptional cascade, resulting in the transcription of 20E primary-response genes that regulate molting and metamorphosis [3]. In all vertebrates, including fish, EcR-USP homologs are the Farnesoid X receptor (FXR) and the Retinoid X receptors (RXR), respectively [3]. FXR is a member of the nuclear receptor 1 (NR1) subfamily that includes the NR1H clade. In fish, it usually contains two or more FXR members [4]. RXR belongs to the NR2 subfamily and constitutes a number of isoforms, including two RXRα, two RXRβ and two RXRγ variants, along with an RXRε and RXRδ that can be observed only in some teleost fish [5,6]. The activity of FXR is also regulated by the heterodimerization with the RXR, due to the permissive nature of the FXR/RXR heterodimer. It has indeed been demonstrated that permissive heterodimers can be directly activated by ligands of one of the two partner receptors [7]. The presence of both ligands can even result in synergistic activation of heterodimers [8]. Among FXR members, FXRα gene is highly conserved from fish to humans, which suggests its involvement in regulating crucial physiological aspects in many animal species [9]. FXR serves as the major transcriptional regulator of bile salt (BA) synthesis and is typically expressed at high levels in the liver and intestine [[10], [11], [12]]. It exerts negative feedback regulation on the synthesis of BAs by inhibiting the rate-limiting enzyme CYP7A1 and maintaining the homeostasis of BAs and cholesterol. In this respect, FXR binds DNA as a homodimer or as heterodimers with RXR and activates the transcription of the short heterodimer partner (SHP) that in turn represses liver receptor homologue-1 (LRH-1)-mediated transcription of CYP7A1 [[13], [14], [15]]. FXR has thus been suggested as a potential target for the treatment of obesity and liver steatosis in mammals [16]. A recent study demonstrates that activation of FXR leads to similar results also in fish [17]. Indeed, FXR was found to play a key role in regulating lipid accumulation in grass carp, since its activation differentially modulated the expression of genes involved in lipid biosynthesis and free fatty acid oxidation, such as sterol regulatory element-binding protein-1c (SREBP-1c) and peroxisome proliferator-activated receptor alpha (PPARα). In recent years, continued research efforts have been focused on finding alternative and more sustainable protein sources for aquafeeds [18,19]. However, some of these dietary fishmeal alternatives, particularly plant-based proteins, have been found to disturb BA status in fish affecting both intestinal reabsorption and/or BA synthesis [20]. More recently, compound feeds used in aquaculture contain increasing amounts of insect meal that has proven to be very promising due to its high-quality protein, balanced amino acid and saturated fatty acid (FA) profiles [21,22]. In this last regard, the inclusion of insect lipids in aquafeed is likely to modulate fish lipid metabolism that is essential for the growth and overall health, particularly in farmed fish. There is evidence that among the mainly studied insect species, the black soldier fly (*Hermetia illucens*) (HI) is one of the most interesting for fishmeal replacement, because it is a good source of protein and bioactive molecules (*e.g*., chitin, antimicrobial peptides, and lauric acid) that can play immunostimulant and/or anti-inflammatory roles in fish [21,23,24]. We recently demonstrated that rainbow trout fed diets containing insect meal obtained from HI prepupae showed the absence of intestinal inflammation due to the presence of bioactive molecules such as lauric acid, tocopherols and carotenoids [22]. However, further data on the effect of fishmeal replacement with insect meal is still required, particularly in relation to endocrine regulation of lipid metabolism. Moreover, to the best of our knowledge, the potential effects of ecdysteroids derived from insect meal on hepatic fat accumulation have never been investigated in teleosts. For that reason, the aim of this study was to evaluate the effects of replacing FM with HIM, on the regulation of lipid and BA homeostasis using rainbow trout (*Oncorhynchus mykiss*) as the animal model. We thus investigated the presence of 20E in a HIM-supplemented diet and assessed the molecular mechanisms/basis of FXR:RXR axis in BA signaling and lipid accumulation.

## 2 Materials and methods

### 2.1 Diet and feeding experiment

Two isoproteic, isolipidic and isoenergetic experimental diets, previously formulated according to the method of Ratti et al., were used in the present study [22]. The first diet, mostly based on marine fish meal and fish oil, was designated as the control (HIM0). The control diet was then reformulated by substituting 3 % of marine ingredients with full fat *Hermetia illucens* to create the second diet (HIM3). The complete chemical composition of diets was reported in Ratti et al. [22]. Rainbow trout juveniles (16.8 ± 3.4 g) were provided by Itticoltura Valpotenza snc (Fiuminata, MC, Italy) and were maintained for a two-week acclimatization period at "Unità di Ricerca e Didattica di San Benedetto del Tronto (AP, Italy), URDIS-University of Camerino". The temperature was maintained at 15.8 ± 0.3 °C and pH values were kept constant at 7.7 ± 0.2. At the beginning of the trial, fish were slightly anesthetized (150 mgL^−1^ of ethyl 3-aminobenzoate methanesulfonate, MS222) and individually measured. Fish were distributed into fiberglass square tanks and fed for 6 weeks, as reported by Ratti et al. [22]. Water quality parameters were monitored every 2 days, showing the following values: pH = 7.5–7.7, O_2_ = 8.0–8.5 ppm and temperature = 13–15 °C; the level of nitrites and ammonia were undetectable. After the exposure, fish were sacrificed with a lethal dose of MS222 (1 g L^−1^) and individually measured. Samples of liver were collected and properly stored at −80 °C prior analyses. Fish manipulation was performed with the ARRIVE guidelines following the procedures established by the European Communities Council Directive for animal health (86/609 and 2010/63) and approved by the local Animal Ethics Committee (Approval Number 6/2021).

### 2.2 RNA extraction and real time PCR

Total RNA was extracted from liver samples (50 mg) using QIAzol Lysis Reagent (Qiagen) according to the manufacturer's instructions. RNA quantification was performed using Qubit RNA High Sensitivity Assay kit together with the Qubit 4.0 Fluorometer (Thermo Fisher Scientific), following guidelines and purity was confirmed by electrophoresis through 1 % agarose gels stained with ethidium bromide. The complementary DNA (cDNA) was synthesized from total RNA (1 μg) in a total volume reaction (20 μL) that contains All-In-One 5X RT MasterMix™ [OneScript® Hot Reverse Transcriptase, RNaseOFF Ribonuclease Inhibitor, temperature-sensitive DNase, dNTPs and a finely-balanced ratio of Oligo (dT) and random primers; abm]. SYBR green-based real-time PCR (q-PCR) was used to evaluate expression profiles of FXR, RXR, SHP-1, SHP-2, RXR, CYP7a, CYP8a, SREBP-1c and PPARα target genes and of the housekeeping gene (*i.e.* 18s rRNA). The specific primers for rainbow trout FXR were designed by using Primer-BLAST software, on the basis of the sequence available at GenBank database (Accession number: AB675939.1) and were as follows: FW- TAGAGGGAGGTATGCTGAGGT and RV-CCTCAACGCTGTATTGGGGG (Efficiency: 93.8 %). All other primer sequences are reported in Table 1. The expression of individual gene targets was analyzed using the ABI7300 Real-time PCR system. Thermo-cycling for all reactions was for 10 min 95 °C, followed by 40 cycles of 15 s at 95 °C and 30 s at 57 °C. Fluorescence was monitored at the end of every cycle, the melting curve and agarose gel electrophoresis confirmed the specificity of primer pairs. Results were calculated using the relative 2^−ΔΔCt^ method [25] and means of mRNA levels are expressed with respect to control fish ± standard deviation (SD).

**Table 1:** Primer pairs used for Real-Time PCR.

Gene	Primers (5’ - 3′)	References	Efficiency (%)
RXR	FW- ACCCGGTGACTAACATCTGC	[26]	94.5
	RV- CAGGAGGATCCCATCTTTCA		
SHP-1	FW- GGAGCTATGCTGTTCAATCCAGACA	[14]	96.3
	RV- GTAAGTCAGAGGTCGATAGTAGGATGCA		
SHP-2	FW- CCAAGAATGGACTGTGTTCGAGGTGAT	[14]	92.8
	RV- CCAGCCAGCGTGGGCAGAG		
CYP7a1	FW- AGGCCAACACGCTCCCGACTG	[14]	95.2
	RV- CCGGGAGAGAGTGAGTTGTGGTTTGCT		
CYP8b1	FW- CACAGTGTAGGGACAAAGCATGATAGAA	[14]	98.7
	RV- CGGGGATTTGGGTGTCTCGTTAC		
PPARα	FW- CTGGAGCTGGATGACAGTGA	[27]	99.6
	RV- GGCAAGTTTTTGCAGCAGAT		
SREBP-1c	FW- CAAGCTGCCCATCAACCGTA	[28]	92.4
	RV- GGCCACCAGGTCTTTAAGCTC		
18s rRNA	FW- GGCGCCCCCTCGATGCTCTTA	[29]	97.6
	RV- CCCCCGGCCGTCCCTCTTAAT		

### 2.3 Total hepatic cholesterol and triglyceride profiles

Lipids were purified and quantified in the liver from the remaining organic phase after Qiazol extraction, as reported by Cocci et al. [30]. Total cholesterol concentration was determined enzymatically by the commercial Cholesterol/Cholesteryl Ester Assay Kit (Abcam) and triglyceride (TG) profile was determined using the commercial Triglyceride Quantification Assay Kit (Abcam), according to the manufacturer's instructions.

### 2.4 Total bile acid (TBA) contents in liver

TBA levels in the supernatant of the homogenized liver were measured using a colorimetric assay (Cell Biolabs Inc.). Optimal experimental conditions for samples have been determined and a set of serial dilutions for samples has been validated to achieve optimal assay results and minimize possible interfering compounds. Briefly sonicated liver samples in cold isopropanol were centrifuged at 10,000×*g* for 10 min at 4 °C. Resulting supernatant was diluted at 5-fold in deionized H_2_O prior to testing in the assay. Changes in absorbance (405/630 nm) of each sample were compared to the standard curve to determine and calculate the quantity of BA present in the sample.

### 2.5 Prediction of FXR response element (FXRE) binding sites

To predict the FXRE-mediated regulation, we carried out an *in silico* analysis using PPARα sequence of *Oncorhynchus mykiss* gene (LOC110521393). We have recognized putative promoters by using the Neural Network Promoter Prediction (NNPP) server [31] (promoter score: 0.8). Then, putative FXRE sequences within promoters were investigated using the NUBIScan tool (Version 2.0; optimal raw score threshold of 0.6). In order to detect FXR sites, two copies of a canonical sequence (AGGTCA) organized as inverted repeats separated by one nucleotide (IR1), everted repeats separated by 8 nucleotides (ER8) or direct repeats separated by four nucleotides (DR4) was chosen for the study of gene sequence [32].

### 2.6 Insect-enriched feed formulation and 20-hydroxy-Ecdysone identification

Samples from both diets (*i.e.* HIM0 and HIM3) and full fat *H. illucens* meal were analyzed with a reverse phase column Luna Omega 3C18 150 × 2.1 mm, 3 mm particle size, protected by a C18 guard column (Phenomenex Inc., USA). Water/methanol elution gradient at a flow rate of 0.2 mL min^−1^. The elution was a linear gradient from 20 % to 60 % MeOH over 30 min, followed by a gradient from 60 % to 100 % in 2 min, followed by isocratic 100 % over 5 min, a linear gradient up to 20 % in 2 min, followed by an equilibration step in 20 % MeOH for 10 min. Detection at 254 nm. MS/MS Spectrometry: Agilent ESI-QToF MS/MS, positive ion mode, collision energy = 20V, [M+H]+.

### 2.7 Molecular docking

The *Oncorhynchus mykiss* farnesoid X receptor sequence (GenBank: BAN16587.1) has been used for the homology modeling (built with ProMod3 3.2.1) [33] using as template the available 3D-structure 4nqa.1.B. QMEANDisCo [34] Global: 0.62 ± 0.05. The obtained model has been validated using Procheck [35]. The *Oncorhynchus mykiss* retinoic acid receptor RXRα-A isoform X1 sequence (XP_036833221.1) has been used for the homology modeling (built with ProMod3 3.2.1) using as template the available 3D-structure 4nqa.2.A. QMEANDisCo Global: 0.70 ± 0.05. The obtained model has been validated using Procheck. The two monomers models of *Oncorhynchus mykiss*, were superimposed on the RXRα-FXR dimer structure of *H. sapiens* (PDB ID: 6A5Y) using the PyMol align function. Ligand 3D-structure has been downloaded from PubChem (PubChem CID: 5459840 https://pubchem.ncbi.nlm.nih.gov/compound/5459840). Molecular Docking has been performed using Autodock Vina 1.1.2 on a 64 bits MacOS platform [36]. The parameters used were: exhaustiveness = 8, center_x = −3.892 Å, center_y = 21.267 Å, center_z = 15.873 Å, size_x = 64.685 Å, size_y = 58.024 Å, size_z = 53.368 Å. 2D representation of the binding site has been obtained by LigPlot + [37].

### 2.8 Statistical analysis

All statistical analyses were undertaken using the GraphPad Prism Software (v. 7.00), (La Jolla, USA; www.graphpad.com). The independent *t*-test was used to compare differences in indexes between the control (HIM0) and HIM3 treatment. All results with p ≤ 0.05 were considered statistically significant.

## 3 Results

### 3.1 Expression of FXR and other genes in the synthetic pathways of BA

**Fig. 1:**
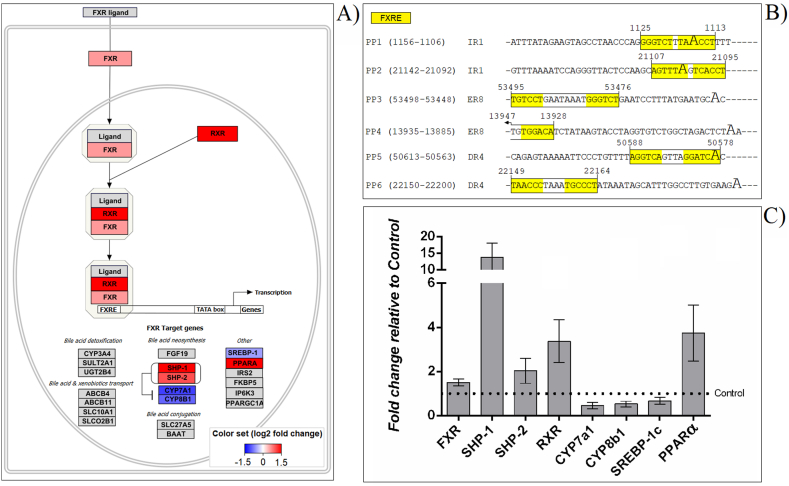
Effects of dietary supplementation with *Hermetia illucens* meal (HIM) on hepatic FXR signaling pathway (WP2879). a) The pathway created was extensively modified from the original pathway to indicate significant (p < 0.05) differentially regulated genes. Coloured squares with the gene name represent the gene expression ratio in the HIM3 fed group with respect to the control diet (HIM0). Up-regulation of genes is indicated by a red coloured box; blue box indicates down-regulation; gray indicates genes that were not investigated. The darker the color, the greater the statistical significance. b) Putative FXRE sequences identified in the PPARα promoters of rainbow trout by using the NUBIScan tool (Version 2.0). c) Fold change in target gene expression in HIM3 fed fish normalized to the internal control gene (18s rRNA) and relative to the control diet (HIM0). Results of the real-time PCR data were represented as mean values ± 95 % confidence intervals.

To explore the ability of 20E, found in HIM-supplemented diets, to activate FXR/RXR axis, we investigated the molecular pathways of BA biosynthesis in the liver. Fish supplemented with HIM showed significantly increased mRNA expression of both FXRα and RXRα. As a consequence, the mRNA levels of their main targets, SHP1 and 2, were also found to be increased by dietary treatments. This latter finding was paralleled by a significant down-regulation of CYP7a1 and CYP8b1 gene expression exclusively in fish from the HIM inclusion group. Finally, PPARα was up-regulated and SREBP-1c was down-regulated in the HIM-supplemented group relative to the control (Fig. 1A–C).

### 3.2 Hepatic levels of cholesterol, triglycerides and bile acids

**Fig. 2:**
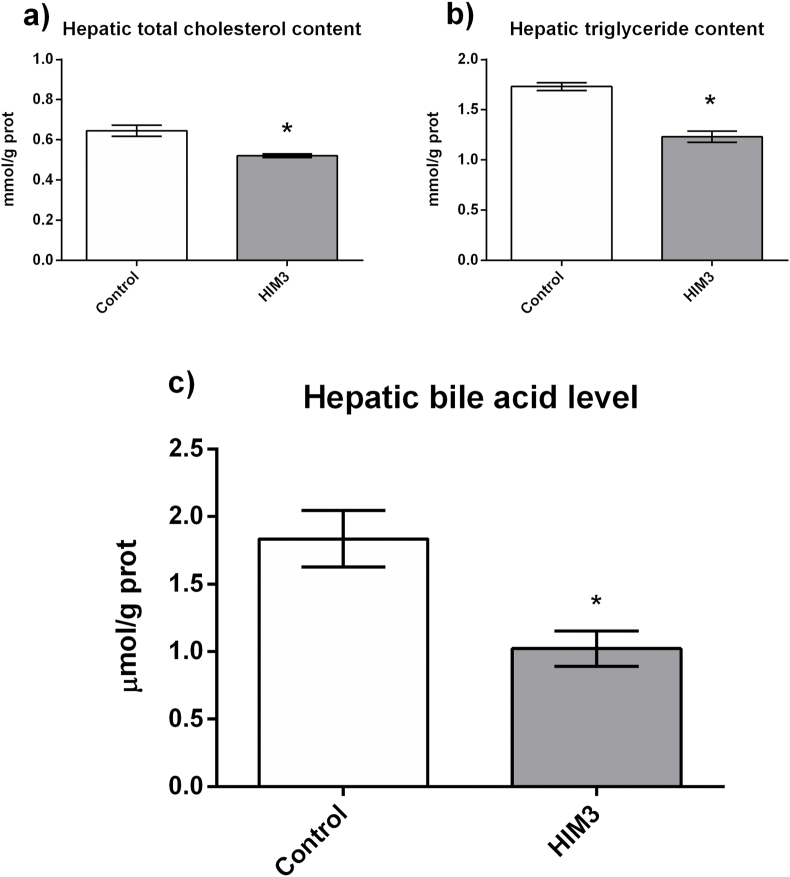
Effects of dietary supplementation with *Hermetia illucens* meal (HIM) on hepatic lipid accumulation and bile acid content of juvenile rainbow trouts. (a) total cholesterol content; (b) triglyceride content; (c) bile acid levels. All results are presented as mean ± standard deviation (SD) (SD is represented by error bars; n = 8). Statistical significance is denoted with asterisks as follows: ∗p < 0.05.

To further confirm the potential role of 20E-induced FXR signaling in the regulation of lipid accumulation, we analyzed the influence of the HIM supplemented diets on hepatic levels of cholesterol, TG and BAs (Fig. 2). Hepatic TG and cholesterol contents were significantly lower in the HIM3 group than in the control group (Fig. 2A and B). The HIM3 group also exhibited decreased BA levels in the liver (Fig. 2C). Collectively, these results suggest that HIM-supplemented diets could alter FXR signaling genes and affect hepatic fat accumulation in rainbow trout.

### 3.3 Identification of 20-hydroxy-Ecdysone in HI-enriched feed formulation and molecular docking

**Fig. 3:**
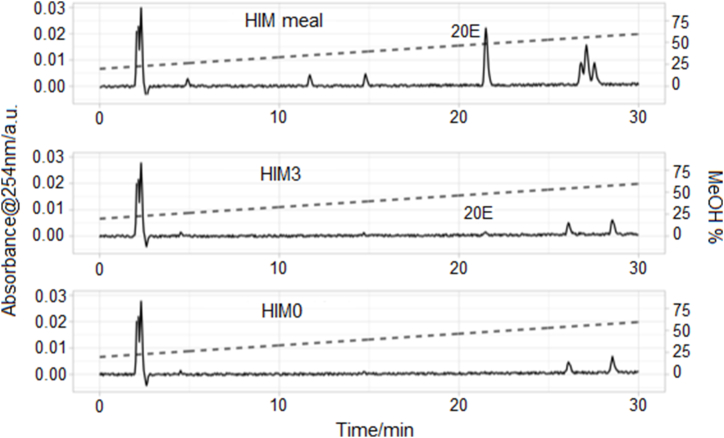
HPLC chromatograms of the analyzed samples. The elution gradient is shown as a dashed line.

**Fig. 4:**
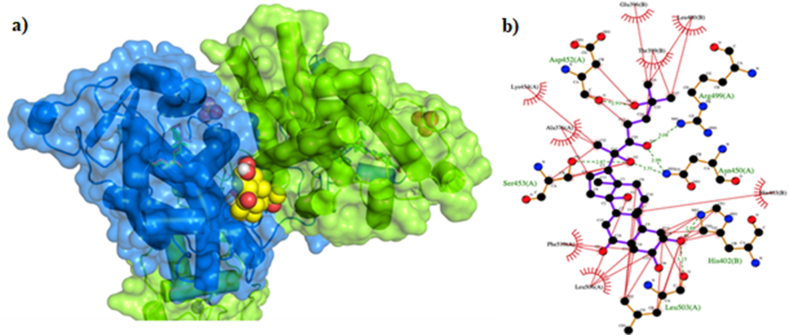
Ligand-receptor interactions. a) Docking best pose; the ligand is shown as vdW spheres, for RXRα (in blue) and FXR (in light green) are shown the solvent-accessible surfaces as calculated using the Connoly algorithm. (b) LigPlot+ 2D schematic representation of the ligand best pose in the heterodimeric receptor binding site. The ligand intramolecular bonds are shown in purple, the receptor amino acids involved in the interaction are shown in black (hydrophobic interactions) and in green (H-bonds). H-bonds are depicted as dashed green lines, together with distances in angstroms.

The parent species (labeled as 20E in Fig. 3) showed *m*/*z* equal to 480.31 (but spiked feed samples also *m*/*z*: 503.3 [M+Na]+). The most abundant fragment showed *m*/*z* equal to 445.29 which is compatible with the fragmentation of 20E (https://pubchem.ncbi.nlm.nih.gov/compound/5459840). The *Oncorhynchus mykiss* heterodimeric FXR-RXRα receptor has been modeled using homology modeling. Molecular docking to predict the interaction between 20E and the *Oncorhynchus mykiss* heterodimeric FXR-RXRα receptor has been performed (Fig. 4). The ligand is able to bind the heterodimeric receptor with a predicted binding affinity constant Kd equals to 610 ± 60 nM. The ligand binds a pocket between the FXR and RXRα subunits, interfering positively on their dimerization (Fig. 4A). The contacts involve hydrophobic moieties and H-bond donors/acceptors with subunit B (FXR: Glu396, Leu460, Thr399, His402, His403) and with subunit A (RXRα: Leu503, Leu506, Phe510, Ser453, Ala376, Lys454, Asp452, Arg499, Asn450) as shown in Fig. 4B.

## 4 Discussion

In our study, we showed that a HIM-supplemented diet increases the mRNA expression of FXR and up-regulates its downstream target genes in the liver. In mammals, the FXR pathway plays crucial roles in maintaining BA homeostasis by regulating the functional connection between liver and intestine [38]. Herein, we focused on the hepatic FXR-SHP pathway. First, our results show that activation of FXR in the liver induces the expression of SHPs, which in turn decrease the mRNA levels of CYP7a1 and CYP8b1 responsible for BA synthesis and BA pool composition, respectively. These findings are largely supported by previous studies reporting the involvement of a regulatory cascade of these genes (*i.e.* FXR, SHP-1) in regulating BA homeostasis [[39], [40], [41]]. However, little is known about the modulation of this signaling pathway in fish. Tian et al. have recently demonstrated that the FXR agonist GW4064 tends to reduce hepatopancreatic total cholesterol and TG accumulation in juvenile grass carp (*Ctenopharyngodon idella*) suggesting the potential role of FXR binding in regulating lipid accumulation in teleost [17]. Accordingly, here we found that HIM-mediated induction of the FXR-SHP pathway reduced hepatic TG, cholesterol and BA levels in rainbow trout. Second, we showed that HIM supplements decreased SREBP-1c mRNA expression. SREBP-1c is a well-known adipogenic transcription factor that preferentially activates genes related to fatty acid and cholesterol synthesis [42,43]. In this regard, it was reported that FXR could reduce hepatic *de novo* synthesis of lipids by modulating the FXR-SHP-SREBP-1c pathway [44]. The molecular mechanism responsible for reducing SREBP-1c expression levels, leading to decreased hepatic TG storage, depends on the FXR-mediated increase of SHP levels. This process exactly resembles the mechanism proposed to explain the reduction of CYP7A1 expression by FXR-SHP, which alters BA homeostasis. In addition, the increased transcription of SHP was found to be followed by a down-regulation of liver X receptor (LXR) suggesting a critical role of LXR as a pivotal regulator of SREBP-1c expression [44]. We conclude that the significant attenuation in the expression of SREBP-1c by SHP induction can explain the inhibitory effects of 20E on BA and TG production. It has been generally accepted that FXR can also control BA homeostasis through crosstalk with PPARα [45] that is likely to play a role especially in terms of BA synthesis and transport. We observed here that the basal transcription levels of PPARα were up-regulated by a HIM-supplemented diet. This is in line with the findings that hPPARα mRNA levels are regulated by BAs *via* a FXRE located within the human PPARα promoter. However, this mechanism has been debated due to its species-specific nature. In this regard, our data demonstrate that the sequence of rainbow trout PPARα contains at least one putative FXRE motif identified as IR1, ER8, DR4 elements (Fig. 1B). We thus speculate that ecdysteroid content of HIM diet may induce FXR and may be responsible for the modulation of PPARα gene transcription. However, it is still controversial whether activation of PPARα results in a reduction of hepatic BAs. On the one hand, PPARα is likely to inhibit BA synthase and the expression of CYP7A1 leading to a down-regulation of BA synthesis [16]. On the other hand, a recent study showed that liver-specific PPARα ligands significantly downregulated SHP mRNA levels, suggesting that PPARα may affect BA synthesis by modulating the FXR-SHP pathway [45]. In addition, Zhou et al. have hypothesized that PPARα competes with FXR for RXR, inhibiting the FXR signaling involved in regulating BA transporters [11]. Besides lowering hepatic content of BA and TG, FXR agonism also lowered hepatic total cholesterol in our study. This finding is consistent with the recent report from Tian et al. in which FXR is an effective target for reducing cholesterol accumulation in the hepatopancreas of grass carp [17]. In contrast, Watanabe et al. found that FXR-SHP mediated down-regulation of CYP7A1 resulted in increased hepatic cholesterol levels in rats [44]. In this regard, we suggest that the persistent decrease in SREBP-1c mRNA levels, observed in our study, would agree with reduced expression of genes involved in cholesterol uptake and biosynthesis. The long-term attenuation of the SREBP-dependent lipogenesis pathway by SHP induction may thus explain the inhibitory effects of FXR agonism on total cholesterol production. Thus, in order to investigate potential agonists for the FXR ligand-binding domain (LBD), contained in HIM supplemented diet, we moved to detect presence of ecdysteroids (i.e. 20E) in the adopted feed formulation. The identification of 20E was performed by combining both untargeted and targeted HPLC-MS/MS approaches that allow to selectively detect the presence of 20E in a complex feed formulation. In insects, ecdysteroids produce genomic effects by binding to specific nuclear receptors of which the equivalent in fish are considered FXR and RXR4. These receptors have a broad specificity and may bind a wide range of xenobiotics, including 20E, working as “endocrine sensors'' [46,47] and mediating transcriptional activity of several key genes involved in BA regulation and lipid homeostasis. Increasing evidence highlights that ecdysteroids have some biological effects in mammals [48] showing very low toxicity [49]. For example, I-hydroxyecdisterone-containing preparations have been found to reduce plasma cholesterol content in rats [50]; however, consequences of ecdysteroid exposure in fish remain largely unknown. Here, we used the available rainbow trout sequence data at first to *in silico* reconstruct the LBD of both FXR and RXRα, and in second to predict the receptor-ligand interactions involved in 20E docking. The obtained 3D model predicts a protein–ligand interaction for 20E that can be considered as a possible binder for both FXR and RXRα monomers. In addition, our results show that 20E forms a stable ternary complex with the FXR and RXR monomers suggesting a critical role in the molecular mechanism of the FXR/RXR heterodimerization. RXR works as a permissive partner for FXR/RXR heterodimer and modulates the FXR regulation of downstream genes resulting in additive or synergistic effects. The molecular mechanisms underlying these effects are likely to be related to conformational changes of the FXR coregulatory-binding site induced by heterodimerization with RXR [51]. This structural mechanism leads to a more effective recruitment of coregulators and transactivation of FXR and enhances its transcriptional activity. Taken together, the results of our analysis support the hypothesis that 20E has a role in FXR/RXR signaling in rainbow trout.

## 5 Conclusions

The present study showed that FXR signaling was activated in liver tissue of rainbow trout fed on an insect-based diet. Further, we investigated the downstream signaling pathways of FXR involved in BA modulation and TG/cholesterol synthesis. Overall, our study suggested that FXR is a potential target for 20E content in insect meal and provided preliminary data on the potential role of ecdysteroids in regulating the metabolic status of teleost fish through the cross-talk between the FXR and PPARα pathways. Nevertheless, due to the high level of complexity in the FXR pathway, further investigation is still needed to clearly identify if agonism of FXR by ecdysteroids causes alterations in other metabolically involved organs, also considering nutrition and health status of fish.

## CRediT authorship contribution statement

**Paolo Cocci:** Writing – review & editing, Writing – original draft, Methodology, Formal analysis, Conceptualization. **Mauro Angeletti:** Writing – review & editing, Writing – original draft, Software, Formal analysis. **Gilberto Mosconi:** Writing – review & editing, Formal analysis. **Ike Olivotto:** Writing – review & editing, Writing – original draft, Validation, Funding acquisition. **Matteo Zarantoniello:** Writing – original draft, Formal analysis. **Francesco Alessandro Palermo:** Writing – review & editing, Writing – original draft, Validation, Software, Methodology, Funding acquisition, Conceptualization.

## Ethics statement

Fish manipulation was performed with the ARRIVE guidelines following the procedures established by the European Communities Council Directive for animal health (86/609 and 2010/63) and previously approved by the local Animal Ethics Committee (Approval Number 6/2021) [22].

## Data availability

Data associated with the study have not been deposited into a publicly available repository, but they will be made available from the corresponding author (F.A.P.) on request.

## Declaration of competing interest

The authors declare that they have no known competing financial interests or personal relationships that could have appeared to influence the work reported in this paper.
